# Nowcasting (Short-Term Forecasting) of Influenza Epidemics in Local Settings, Sweden, 2008–2019

**DOI:** 10.3201/eid2611.200448

**Published:** 2020-11

**Authors:** Armin Spreco, Olle Eriksson, Örjan Dahlström, Benjamin John Cowling, Matthew Biggerstaff, Gunnar Ljunggren, Anna Jöud, Emanuel Istefan, Toomas Timpka

**Affiliations:** Linköping University Department of Health, Medicine, and Caring Sciences, Linköping, Sweden (A. Spreco, E. Istefan, T. Timpka);; Center for Health Services Development, Region Östergötland, Linköping (A. Spreco, T. Timpka);; Linköping University Department of Computer and Information Science, Linköping (O. Eriksson, T. Timpka);; Linköping University Department of Behavioral Sciences and Learning, Linköping (Ö. Dahlström);; World Health Organization Collaborating Centre for Infectious Disease Epidemiology and Control, The University of Hong Kong School of Public Health, Hong Kong (B.J. Cowling);; Centers for Disease Control and Prevention, Atlanta, Georgia, USA (M. Biggerstaff);; Karolinska Institutet Department of Neurobiology, Care Sciences, and Society, Huddinge, Sweden (G. Ljunggren);; Public Health Care Services Committee Administration, Region Stockholm, Stockholm, Sweden (G. Ljunggren);; Lund University Faculty of Medicine, Department of Laboratory Medicine, Division of Occupational and Environmental Medicine, Lund, Sweden (A. Jöud);; Lund University Faculty of Medicine, Clinical Sciences, Division of Orthopedics, Lund (A. Jöud);; Scania University Hospital Department for Research and Development, Lund (A. Jöud)

**Keywords:** influenza, viruses, Sweden, respiratory diseases, vaccine-preventable diseases, epidemiology, infectious disease, human influenza, modelling, signal detection analysis, surveillance, evaluation research, respiratory infections

## Abstract

The timing of influenza case incidence during epidemics can differ between regions within nations and states. We conducted a prospective 10-year evaluation (January 2008–February 2019) of a local influenza nowcasting (short-term forecasting) method in 3 urban counties in Sweden with independent public health administrations by using routine health information system data. Detection-of-epidemic-start (detection), peak timing, and peak intensity were nowcasted. Detection displayed satisfactory performance in 2 of the 3 counties for all nonpandemic influenza seasons and in 6 of 9 seasons for the third county. Peak-timing prediction showed satisfactory performance from the influenza season 2011–12 onward. Peak-intensity prediction also was satisfactory for influenza seasons in 2 of the counties but poor in 1 county. Local influenza nowcasting was satisfactory for seasonal influenza in 2 of 3 counties. The less satisfactory performance in 1 of the study counties might be attributable to population mixing with a neighboring metropolitan area.

Reliable forecasts of the timing and spatial spread of influenza during seasons and pandemics can meaningfully advance the timing of public health communication campaigns and implementation of resource allocation in healthcare (*1*). Different types of influenza forecast methods have been developed and applied to support public health response (*2*). However, although modelers have shown considerable interest in developing infectious disease forecasts, the readiness in the public health community for applying these predictions has been lacking (*3*). One reason for this discrepancy might be that national public health policies for response to infectious disease outbreaks often assign the responsibility for healthcare resource allocation to local health authorities (i.e., county and municipality governments). For geographic and infrastructural reasons, the timing of the spatial spread of influenza can differ substantially between these administrative units within nations and states. Therefore, a need exists for influenza forecasting methods that harmonize with policy-making responsibilities at local government levels and that are more relevant for public health practitioners.

Another reason for the poor uptake of forecasting methods might be a lack of prospective evaluations of their reliability. To address this issue, the US Centers for Disease Control and Prevention (CDC) has run the Forecast the Influenza Season Collaborative Challenge (FluSight) since the 2013–14 influenza season to prospectively evaluate different methods and data sources for influenza forecasting at the national, regional, and (starting in the 2017–18 influenza season) state level (*4*). At the local (county and municipality) level, however, few corresponding prospective evaluations based on routine health system data have been reported. Short-term forecasting is denoted as nowcasting (*5*). Recently, a prospective 5-year appraisal of a local nowcasting method (*6*) in a county in Sweden (county population ≈460,000) indicated promising results with regard to detection of the local start of the epidemic, prediction of peak timing, and prediction of peak intensity (*7*). The appraisal concluded that a longer prospective evaluation was needed to ascertain the validity of the results and that data from larger urban counties were required to draw reliable conclusions about generalizability.

In this article, we describe a prospective 10-year evaluation of this local influenza nowcasting method in 3 urban counties (population 1.3–2.2 million) in Sweden. The evaluation period included 1 pandemic (2009) and 9 seasonal influenza epidemics.

## Methods

### Study Design

**Figure 1:**
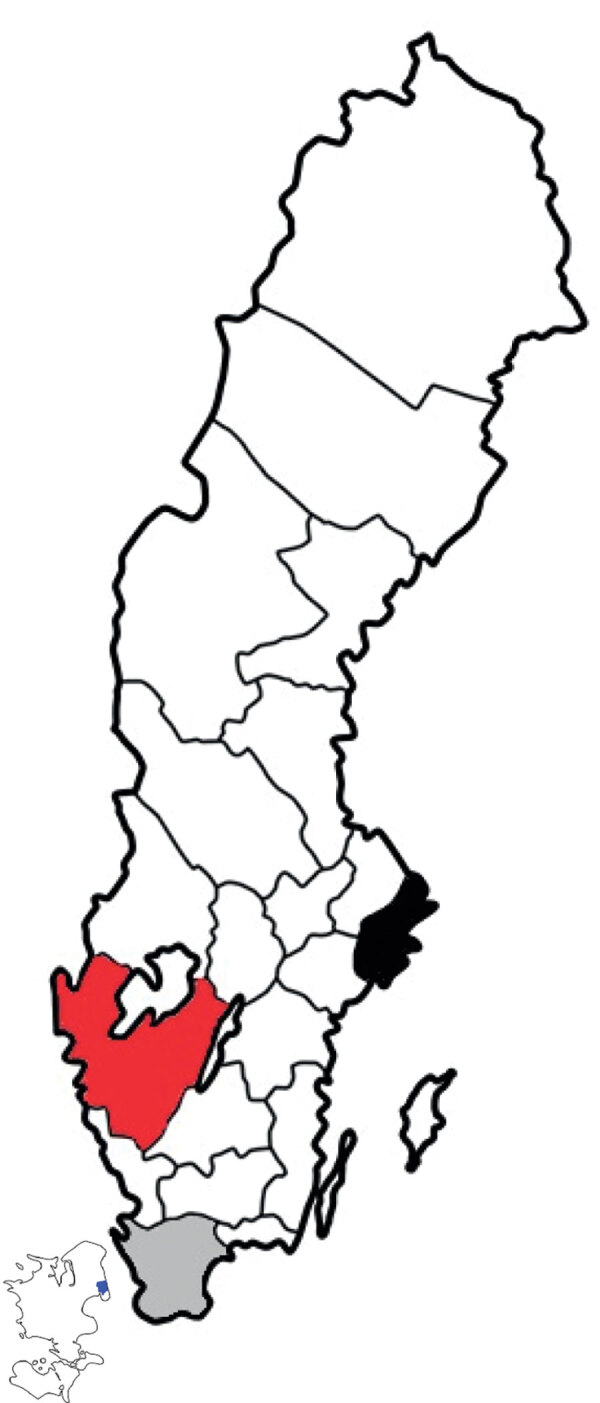
Three regions analyzed in study of nowcasting for influenza epidemics in local settings, Sweden. Black indicates Stockholm County, red West Gothia County, gray Scania County. Included in the map is the island Zeeland (Sjaelland) (which is neighboring to Scania County). Blue indicates the city of Copenhagen (population 2 million) (on the island in the left lower corner of the figure).

**Figure 2:**
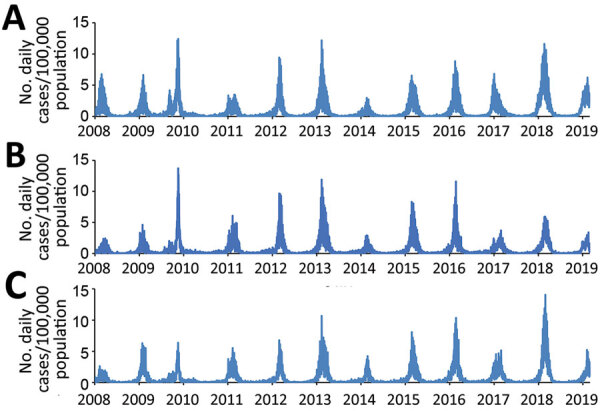
Daily numbers of influenza-diagnosis cases per 100,000 population, January 1, 2008–February 28, 2019, in Stockholm County (upper graph), West Gothia County (middle graph), and Scania County (lower graph), Sweden.

**Figure 3:**
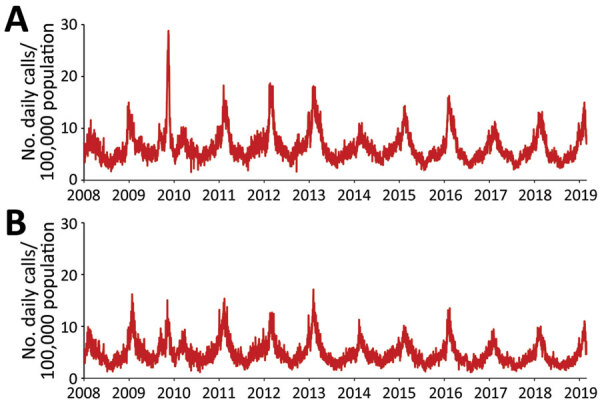
Daily numbers of telenursing calls attributable to fever (among children and adults) per 100,000 population, January 1, 2008–February 28, 2019, in West Gothia County (upper graph) and Scania County (lower graph), Sweden.

We used an open cohort design based on the total population in 3 urban counties: Stockholm County (population 2,231,000), West Gothia County (population 1,649,000), and Scania County (population 1,304,000) (Figure 1). We used retrospective data from January 1, 2008, through June 30, 2009, and prospective data from July 1 through February 28, 2019, from 2 sources in the countywide health information system: daily numbers of clinically diagnosed influenza cases (Figure 2) and daily syndromic chief complaint data from a telenursing service (Figure 3) (*6*,*7*). The clinical influenza case data were used for detection of the local start of the epidemic and prediction of its peak intensity, and the syndromic data were used to detect the peak timing. Existing evidence of to predict strong association between the clinical influenza case data and syndromic chief complaint data from the telenursing service was used in this nowcasting method (*8*,*9*). Because of a change of system, no syndromic chief complaint data were available for Stockholm County. Syndromic data from West Gothia County were therefore used to predict of the peak timing for Stockholm County.

Timeliness was used as a performance metric for detection of the local start of the epidemic and the peak-timing prediction; the correct identification of intensity category on a 5-grade scale (i.e., nonepidemic, low, medium, high, and very high) was used for peak-intensity prediction. The study design was approved by the Regional Research Ethics Board in Linköping (approval no. 2012/104-31).

### Definitions

Influenza cases were identified by using codes from the International Classification of Diseases, 10th Revision, for influenza (J10.0, J10.1, J10.8, J11.0, J11.1, J11.8) (*10*) as recorded in the local electronic health data repository. Only influenza diagnoses in the first coding position were used. Influenza-related telenursing call cases were identified by using the chief complaint codes associated with influenza symptoms. The symptoms used were fever, cough, and headache. These data were downloaded from the electronic patient record systems to the electronic health data repository twice daily.

The intensity level defining the start of a local epidemic (i.e., the intensity that determines the endpoint for the detection function) was set to 6.3 influenza-diagnosis cases/100,000 population recorded during a floating 7-day period in the countywide health information system (*6*). This level was chosen by inspecting the epidemic curves of previous local influenza epidemics. A recent comparison of influenza intensity levels in Europe estimated a similar level (6.4 influenza-diagnosis cases/week/100,000 population) for the 2008–09 seasonal influenza in Sweden (*11*). The optimal alerting threshold before each epidemic was decided by calculating the sensitivity and the specificity for the previous nonpandemic influenza seasons and studying them on a receiver operating characteristic curve (*6*). The calculation of the specificity was based on all days in the nonepidemic period (i.e., before the limit of 6.3 influenza-diagnosis cases/100,000 during a floating 7-day period occur), and the calculation of the sensitivity was based on the days in the epidemic period (i.e., from when the limit of 6.3 influenza-diagnosis cases/100,000 during a floating 7-day period has occurred). Peak timing was defined as the day when the highest number of influenza-diagnosis cases were documented in the countywide electronic patient record. Peak intensity was defined as the number of influenza-diagnosis cases that had been documented at peak timing.

### Method Application

Technical details concerning the 3 functions of nowcasting have been described previously (*6*; Appendix). The functions are detecting the start of the influenza season or pandemic and forecasting the peak day and peak intensity. Once the epidemic has been detected using the clinical influenza data, the syndromic telenursing data are used to detect when it decreases, that being the indication for the peak. Because changes in clinical influenza data have been found to occur 14 days after corresponding changes in syndromic data, the peak timing in the clinical influenza data are forecasted to occur 14 days after the peak in the syndromic data. Finally, the peak intensity is forecasted by using the clinical influenza data. Syndromic data have a higher amplitude, and the relationship between syndromic data and clinical influenza data are not necessarily constant between seasons. Therefore, the clinical data were used to predict the intensity once the peak day is predicted with the help of syndromic data.

To calibrate the detection component of the nowcasting method, we retrospectively determined weekday effects on recording of influenza-diagnosis cases and a baseline alert threshold by using the retrospective data. These data were collected from January 1, 2008, through June 30, 2009, including 2 nonpandemic influenza seasons (2007–08 and 2008–09). To determine weekday effects, data from the entire retrospective data collection period were used. To determine the initial alert threshold, only data from the seasonal influenza in 2008–09 were used. The 2007–08 seasonal influenza could not be used for this purpose because the season had started before January 1, 2008. Throughout the study period, the calibration data were updated after every seasonal influenza (i.e., no updates of the threshold after the 2009 pandemic outbreak). The detection algorithm was thus applied to the next epidemic by using the revised threshold determined in the updated retrospective dataset.

Before the 2010–11 seasonal influenza, no updates were performed because the set of retrospective data remained the same (i.e., it contained data from the 2008–09 seasonal influenza but excluded pandemic data). For the 2011–12 seasonal influenza, the threshold was updated by using retrospective data from the 2008–09 and 2010–11 seasonal influenza. For the 2012–13 seasonal influenza, the threshold was updated by using retrospective data from the 2008–09, 2010–11, and 2011–12 seasonal influenza, and so on. The weekday effects were assumed to be relatively constant over time in the local detection analyses and were therefore not updated after every seasonal influenza.

The set of retrospective data from the seasonal influenza in 2007–08 and in 2008–09 were also used to initially calibrate peak-timing prediction for West Gothia County and Scania County. The dataset was used to decide the grouping of chief complaints with the largest correlation strength and longest lead time from telenursing data to influenza-diagnosis data (*10*,*11*). For both counties, the best performing telenursing chief complaint was fever (among children and adults), and the most favorable lead time was 14 days. When the peak timing had been determined, the second component of the local prediction module was applied to influenza-diagnosis data from the corresponding epidemics to find the peak intensity on the predicted peak day (*6*). Regarding weekday effects on local prediction, the same calculation was applied and the same grouping of chief complaints and lead time were used throughout the study.

### Metrics and Interpretations

On the basis of the utility of the nowcasting method in local healthcare settings, the maximum tolerable timeliness error for detection and peak-timing predictions was set to 11 days (»1.5 weeks). Method performance was defined to be excellent if the absolute value of the timeliness error was <3 days, good if it was 4–7 days, tolerable if it was 8–11 days, and poor if it was >12 days. For the interpretation of peak intensity predictions, the intensity level categories (nonepidemic <0.9, low 0.9, medium 2.4, high 5.5, and very high intensity level 7.9 cases/day/100,000 population) identified using the moving epidemic method for the reference influenza season 2008–09 in Sweden (*11*) were used. If the predicted peak intensity fell into the same category as the recorded peak intensity, the prediction was considered excellent. If the predicted peak intensity did not fall into the same intensity category, the predicted peak was considered good if it was up to 10% above or below the threshold for the recorded peak intensity category, tolerable if the predicted peak was 10%–20% above or below the threshold for the recorded peak intensity category, and poor otherwise. When assessing series of nowcasts, the performance of a sequence of nowcasts was considered satisfactory if all separate forecasts were assessed as excellent, good, or tolerable, and poor otherwise.

## Results

### Local Detection

The date of the actual start of the epidemic phase for the 10 influenza epidemics differed by 2–27 days between the 3 counties (Table 1). The detection component of the local nowcasting method showed good or excellent performance in all counties under surveillance for 6 of the 9 nonpandemic influenza seasons and in 2 out of 3 counties under surveillance for the 3 remaining seasons. Twice the poor alerts were issued too soon and once belatedly. The detection performance was good during the 2009 influenza A(H1N1) pandemic in 2 of 3 counties (Stockholm and West Gothia) and poor in 1 county (Scania).

**Table 1:** Performance of the detection algorithm displayed with alert thresholds updated by using data from previous nonpandemic influenza seasons in evaluation of nowcasting for detection and prediction of local influenza epidemics, Sweden, 2008–2019

Influenza virus activity	Updated* alert threshold, cases/day/100,000 population†	Timeliness‡	Start according to method	Actual start§	Interpretation
2008–09 A(H3N2), initial retrospective data					
Stockholm	0.63				
West Gothia	0.73				
Scania	0.25				
2009 A(H1N1)					
Stockholm	0.63	−5	2009 Aug 24	2009 Aug 19	Good
West Gothia	0.73	−6	2009 Sep 3	2009 Aug 28	Good
Scania	0.25	18	2009 Aug 13	2009 Aug 31	Poor
2010–11 A(H1N1) and B¶					
Stockholm	0.63	−7	2010 Dec 30	2010 Dec 23	Good
West Gothia	0.73	−12	2011 Jan 9	2010 Dec 28	Poor
Scania	0.25	2	2010 Dec 23	2010 Dec 25	Excellent
2011–12 A(H3N2)					
Stockholm	0.59	2	2012 Jan 22	2012 Jan 24	Excellent
West Gothia	0.43	1	2012 Jan 31	2012 Feb 1	Excellent
Scania	0.27	23	2012 Jan 9	2012 Feb 1	Poor
2012–13 A(H3N2), A(H1N1), and B					
Stockholm	0.51	−6	2013 Jan 3	2012 Dec 28	Good
West Gothia	0.44	0	2012 Dec 29	2012 Dec 29	Excellent
Scania	0.28	0	2012 Dec 27	2012 Dec 27	Excellent
2013–14 A(H3N2), A(H1N1), and B					
Stockholm	0.52	0	2014 Jan 30	2014 Jan 30	Excellent
West Gothia	0.37	1	2014 Jan 27	2014 Jan 28	Excellent
Scania	0.35	0	2014 Jan 28	2014 Jan 28	Excellent
2014–15 A(H3N2) and B					
Stockholm	0.52	−6	2015 Jan 13	2015 Jan 7	Good
West Gothia	0.39	0	2015 Jan 17	2015 Jan 17	Excellent
Scania	0.35	7	2015 Jan 16	2015 Jan 23	Good
2015–16 A(pH1N1) and B					
Stockholm	0.52	0	2016 Jan 2	2016 Jan 2	Excellent
West Gothia	0.47	16	2015 Dec 28	2016 Jan 13	Poor
Scania	0.34	0	2015 Dec 16	2015 Dec 16	Excellent
2016–17 A(H3N2)					
Stockholm	0.34	−2	2016 Dec 1	2016 Nov 29	Excellent
West Gothia	0.31	−2	2016 Dec 17	2016 Dec 15	Excellent
Scania	0.31	0	2016 Dec 10	2016 Dec 10	Excellent
2017–18 A(H3N2) and B					
Stockholm	0.38	0	2017 Dec 12	2017 Dec 12	Excellent
West Gothia	0.44	4	2017 Dec 30	2018 Jan 3	Good
Scania	0.34	5	2017 Dec 22	2017 Dec 27	Good
2018–19 A(pH1N1)					
Stockholm	0.36	−7	2018 Dec 18	2018 Dec 5	Good
West Gothia	0.40	−6	2018 Dec 28	2018 Dec 22	Good
Scania	0.34	5	2018 Dec 27	2019 Jan 1	Good

*Threshold updated after every seasonal influenza (i.e., no updates after pandemic outbreaks). †Threshold determined using clinical influenza-diagnosis data. ‡Positive value means that the algorithm issued an alarm before the local epidemic had started; negative value means that the alarm was raised after the start of the epidemic. §Actual start is the date when the retrospectively calculated intensity level reached the predefined threshold for start of an epidemic (6.3 influenza-diagnosis cases/100,000 population recorded during a floating 7-day period) (*7*,*11*). ¶No update of threshold before this seasonal influenza because the previous outbreak was a pandemic.

### Local Prediction

For the 2009 influenza pandemic, the performance of the peak-timing prediction was poor in all 3 study counties (Table 2). The peak-timing prediction was also poor for the 2010–11 seasonal, when influenza A(H1N1) and B viruses were circulating. Thereafter, the predictions were tolerable for the 2011–12 seasonal influenza, when influenza A(H3N2) virus was circulating, and good to excellent for the remaining influenza seasons, with the exception of the poor peak-timing predictions for Scania County for the 2016–17 and 2017–18 influenza seasons, with influenza A(H3N2) virus circulating in 2016–17 and influenza A(H3N2) and B in 2017–18.

**Table 2:** Performance of peak-timing and peak-intensity predictions from evaluation of nowcasting for detection and prediction of local influenza epidemics, Sweden, 2008–2019

Influenza virus activity	Time-to-peak*					Peak-intensity category, cases/day/100,000 population†§		
	Prediction date	Predicted	Error	Interpretation		Predicted	Factual	Interpretation
2009 A(H1N1)								
Stockholm	2009 Sep 13	8	56	Poor		Medium (5.0)	Very high (12.4)	Poor
West Gothia	2009 Sep 13	8	56	Poor		Low (2.2)	Very high (13.7)	Poor
Scania	2009 Sep 25	10	42	Poor		Low (1.4)	High (6.4)	Poor
2010–11 A(H1N1) and B								
Stockholm	2011 Jan 14	10	28	Poor		Medium (3.4)	Medium (3.5)	Excellent
West Gothia	2011 Jan 14	10	14	Poor		Medium (4.3)	High (6.1)	Tolerable
Scania	2011 Jan 10	11	22	Poor		Medium (2.9)	High (5.5)	Poor
2011–12 A(H3N2)								
Stockholm	2012 Feb 27	8	−8	Tolerable		High (7.4)	Very high (9.4)	Good
West Gothia	2012 Feb 27	8	−8	Tolerable		High (7.8)	Very high (9.6)	Good
Scania	2012 Feb 27	8	−8	Tolerable		Medium (4.0)	High (6.8)	Poor
2012–13 A(H3N2), A(H1N1), and B								
Stockholm	2013 Feb 10	8	−7	Good		Very high (10.3)	Very high (12.2)	Excellent
West Gothia	2013 Feb 10	8	−7	Good		Very high (10.3)	Very high (11.9)	Excellent
Scania	2019 Feb 8	10	−7	Good		High (7.3)	Very high (10.7)	Good
2013–14 A(H3N2), A(H1N1), and B								
Stockholm	2014 Feb 16	8	−7	Good		Medium (2.7)	Medium (3.0)	Excellent
West Gothia	2014 Feb 16	8	−7	Good		Medium (3.5)	Medium (2.9)	Excellent
Scania	2014 Feb 17	8	−1	Excellent		Medium (3.2)	Medium (4.2)	Excellent
2014–15 A(H3N2) and B								
Stockholm	2015 Feb 22	8	6	Good		Medium (4.5)	High (6.5)	Tolerable
West Gothia	2015 Feb 22	8	6	Good		Very high (7.9)	Very high (8.3)	Excellent
Scania	2015 Feb 14	9	0	Excellent		Medium (3.9)	Very high (8.1)	Poor
2015–16 A(H1N1) and B								
Stockholm	2016 Feb 7	8	0	Excellent		High (6.7)	Very high (8.2)	Tolerable
West Gothia	2016 Feb 7	8	7	Good		High (7.6)	Very high (11.6)	Good
Scania	2016 Feb 6	9	7	Good		Medium (4.3)	Very high (10.4)	Poor
2016–17 A(H3N2)								
Stockholm	2017 Jan 1	8	−7	Good		Very high (8.2)	High (6.8)	Good
West Gothia	2017 Feb 12	8	7	Good		Medium (3.3)	Medium (3.7)	Excellent
Scania	2017 Feb 5	8	14	Poor		Medium (4.2)	Medium (5.1)	Excellent
2017–18 A(H3N2) and B								
Stockholm	2018 Feb 18	8	−7	Good		Very high (14.4)	Very high (11.6)	Excellent
West Gothia	2018 Feb 18	8	0	Excellent		Medium (5.2)	High (5.9)	Good
Scania	2018 Feb 4	8	14	Poor		Medium (4.2)	Very high (14.0)	Poor
2018–19 A(H1N1)								
Stockholm	2019 Feb 3	8	0	Excellent		Very high (14.4)	High (6.2)	Poor
West Gothia	2019 Feb 3	8	7	Good		Medium (4.0)	Medium (3.4)	Excellent
Scania	2019 Feb 3	8	−7	Good		Medium (2.8)	Medium (5.2)	Excellent

*Time-to-peak (days) determined using syndromic telenursing data. Positive value means that the peak was predicted to be reached before the actual peak occurs, whereas negative value means that the peak is predicted after the actual peak occurs. †Peak-intensity category determined using clinical influenza-diagnosis data. §Using clinical influenza data (Table 1), the start of the epidemic was detected on December 27. On February 1, using syndromic data, the peak in clinical influenza data was forecasted to occur 8 days later (February 9), but the peak actually occurred on February 2 (7 days earlier than forecasted). Also, on February 1, the clinical influenza data intensity was forecasted to be high.

The prediction of the peak-intensity level was poor for the 2009 influenza pandemic in all 3 study counties (Table 2). For seasonal influenza, in 2 of the study counties (Stockholm and West Gothia) the predictions were tolerable to excellent for all seasons, except for the 2018–19 season with influenza A(H1N1) in Stockholm. In 1 county (Scania), the peak-intensity predictions were poor for 5 of the 9 influenza seasons: 2010–11 with influenza A(H1N1) and B, 2011–12 with influenza A(H3N2), 2014–15 with influenza A(H3N2) and B, 2015–16 with influenza A(H1N1) and B, and 2017–18 with influenza A(H3N2) and B circulating.

## Discussion

Epidemic forecasts for large administrative areas (e.g., nations or states) might not be sufficiently informative for local response to epidemics if sizable variations in disease transmission patterns exist between the smaller administrative areas (e.g., counties) with independent local healthcare governance that they contain (*12*). The importance of taking the local context into regard in epidemic forecasting has been further emphasized during the current coronavirus pandemic (*13*). In our prospective 10-year evaluation of local nowcasting in 3 urban counties, the start of the influenza seasons included differed by up to 27 days and the peak intensity by >1 intensity level among the counties, whereas the time-of-peak differences were small. The purpose of the evaluated local detection function was to allow hospitals and primary healthcare centers time to prepare for management of influenza patients (e.g., by preparing intensive care unit resources or postponing some elective procedures). This component showed satisfactory performance in all 3 counties. The peak-timing prediction function was aimed at informing the local authorities when the peak has occurred and that health service routines soon can be permitted to return to normal. This component showed satisfactory performance from the 2011–12 influenza season onward. Predictions of peak timing were made 8–10 days before the peak and were +7 days accurate in most cases. This finding contrasts with the current practices in the study counties, where the peak of an influenza season is retrospectively determined from surveillance data »10–14 days after it has occurred. The nowcasting of peak-intensity level was aimed at warning the local authorities about high-intensity influenza transmission and the potential need for social distancing measures (e.g., closure of kindergartens). This component provided satisfactory information for influenza seasons in 2 out of 3 study counties (Stockholm and West Gothia).

Although the evaluated nowcasting method is automated to run on routinely collected healthcare data, the accuracy of the nowcasts depends on the stability of the data supply and information infrastructure over time. The method does not require influenza cases to be confirmed by a laboratory as long as data recording remains relatively stable. Nonetheless, some observations can be made about the sensitivity of the local nowcasts to contextual factors. In Sweden, vaccination adapted to the current circulating strains is made available free-of-cost to the elderly and risk groups before every influenza season. However, in the case of the 2009 influenza A(H1N1) pandemic, a national vaccination campaign was implemented, covering the entire population. This intervention probably influenced the nowcasting performance during the corresponding period. Looking only at the performance for seasonal influenza, we observed outcomes in 1 of the 3 study counties (Scania) the raise concerns about vulnerability of the nowcasts to sociodemographic dynamics (*14*). Malmö (population 450,000; capital of Scania County, Sweden) and Copenhagen (population 2 million; capital of Denmark) are connected by a bridge providing for daily commuting between the metropolitan areas, and their labor markets are closely integrated. The epidemic situation in the highly cosmopolitan Copenhagen region might have had a stronger influence on influenza epidemics in Scania County than the epidemic situation in the neighboring regions had on the other study counties. By structured introduction, evaluation, and modification of prediction models that use additional data sources and statistical methods, local nowcasting can be adapted also to communities with unusual characteristics (*15*,*16*). This evidence-based strategy means that, our method can be incrementally adapted to modeling of, for instance, local rural or semirural communities in which residents commute extensively to a neighboring city that is not included in the model.

Some possible limitations exist in terms of the design of this prospective evaluation that require attention. First and foremost, whether the framework used to interpret the nowcasting performance is adequate from the local health authority perspective should be assessed. Regarding the time-of-peak predictions, the ongoing FluSight study uses weekly data (*4*), thus accepting forecasts made at a weekly resolution. The evaluation framework used to classify forecasts as excellent was at a higher temporal resolution (less than one half week). This boundary was defined from a county government perspective, where the attention is on local resource allocation (e.g., intensive-care unit facilities and hospital beds) for the care of influenza patients. In this situation, nowcasts that are off by days to weeks might have severe consequences for patients in need of these resources. Categories that are suitable for evaluation of usefulness in local response preparations might not be suitable for interpretation of utility in national or international response planning. These observations suggest that the requirements on the accuracy of peak-timing predictions are context-dependent and warrant further research. Concerning the predictions of peak intensity, evaluation of the peak-intensity forecasts indicated that 22% (6 of 27) of the seasonal influenza nowcasts were poor. Retrospectively documenting baseline and threshold values for influenza epidemics helps define whether an influenza epidemic has been different in intensity compared with previous seasons and thereby contributes to future preparedness planning (*17*,*18*). For the evaluation of intensity predictions in this study, we used the thresholds established using the moving epidemic method from the reference 2008–09 seasonal influenza season. To improve the validity of the assessments, annual updates of the threshold values using county-level data from previous seasons should be considered for future evaluations of local influenza nowcasting.

Longitudinal prospective evaluations might be needed to draw valid conclusions concerning the performance of local epidemic nowcasting, and inclusion of data from urban counties might be required for generalizability (*7*). We found in our study that the performance of seasonal influenza nowcasting was satisfactory during a 10-year period in 3 urban counties regarding local detection and peak-timing prediction performance. The predictions of the local peak-intensity level were satisfactory in 2 of the study counties but poorer in 1 county, possibly because of sudden sociodemographic changes. We conclude that the performance of the local nowcasting method was satisfactory for seasonal influenza. The results are of general interest for local healthcare planning during epidemics because the precision by which healthcare systems can adapt its resources to the management of infected patients in these situations affects the resource availability for all other patient groups.

Appendix Additional information about nowcasting (short-term forecasting) of influenza epidemics in local settings, Sweden, 2008–2019.
